# Gradient magnetometer dataset and MATLAB numerical code for simulating buried firearms at a controlled field site

**DOI:** 10.1016/j.dib.2020.106050

**Published:** 2020-07-21

**Authors:** Kennedy O. Doro, Elijah Achuoth Deng, Carl-Georg Bank

**Affiliations:** a Department of Environmental Sciences, University of Toledo, 2801 West Bancroft Street, Toledo, Ohio, 43606-3390, USA; b Department of Earth Sciences, University of Toronto, 22 Russell Street, Toronto, Ontario M5S3B1, Canada

**Keywords:** Forensic geophysics, Magnetic gradiometry, Controlled geophysical research, Detecting buried weapons, MATLAB numerical code

## Abstract

Magnetic survey using multiple magnetometers to obtain gradiometric data can be used as a non-destructive method to search for buried firearms. We present magnetic dataset collected above a set of weapons buried at 0.6 m, 1.2 m, and 1.8 m depths. We provide three datafiles: two datafiles were collected on a coarse grid (1 m by 0.5 m) before and after burial of the weapons, and a third one collected on a fine grid (0.25 m by 0.1 m) after the burial of the weapons which concentrates on the area of buried firearms. We used a Gem Systems GSM-19GW Overhauser gradiometer consisting of two sensors with a relative vertical separation of 55 cm. Data acquisition was done via non-automated point measurements within a gridded measurement domain with data collection locations managed using measurement tape. Each field campaign resulted in about 3,000 data points. In addition, we developed a set of MATLAB scripts to model the magnetic anomalies (total field and gradient) for buried firearms, this set is also included here. The data and modeling scripts relate to a research article published in Forensic Science International (Deng et al., Suitability of magnetometry to detect clandestine buried firearms from a controlled field site and numerical modeling [1]). The dataset may be helpful for testing new algorithms for weapons detection while the numerical codes can be modified and applied for simulating magnetic anomalies resulting from similar buried objects with potential application in the sub-disciplines of forensic and archaeological geophysics.

Specifications Table

**Table:** 

Subject	Geophysics
Specific subject area	Near-surface geophysics, forensic geophysics (non-destructive search for buried firearms using magnetic gradiometer).
Type of data	Tables (as text files) Figures MATLAB scripts Note on reading data and running the MATLAB scripts
How data were acquired	Magnetic gradiometer field data were acquired using a Gem System GSM-19GW Overhauser gradiometer consisting of 2 sensors with a relative vertical separation of 55 cm. Point measurements were recorded every 0.5 m step with a line spacing of 1 m for the coarse grid data set prior to and after burial of the weapons and at every 0.1 m step with a line spacing of 0.25 m for the fine grid survey after burial of the weapons. Magnetic base station data were measured using Gem Systems GSM-19T and Geometrics G856, both proton precession magnetometers. Our MATLAB scripts were written following equations developed by Seleznyyova et al [2], [3], [4]. We converted the cylindrical coordinates presented by the authors based on distances of measurement points to the centre of the dipole and their angles into a cartesian coordinates (North, East and vertical dip) using applicable vector algebra [1]. We also used the superposition principle to obtain vector sum of the earth magnetic field and that resulting from the buried weapons [1].
Data format	Raw (ASCII files downloaded from magnetometer) Analyzed (MATLAB mat files)
Parameters for data collection	Magnetic gradiometer sensors were at 25 and 80 cm height above ground (i.e. with a relative vertical spacing of 55 cm). Instrument sensitivity: 0.022nT / √Hz Instrument Accuracy: +/- 0.1 nT Operation mode: manual with data acquisition following established sampling grid (0.5 m by 1 m for coarse grid and 0.25 m by 0.10 m for fine grid)
Description of data collection	The test site where these datasets were acquired contains handguns and rifles buried at 0.6 m, 1.2 m and 1.8 m depths (Fig. 1). Magnetic data were collected in 3 different campaigns in June 202 (prior to weapon burial), August 2012 (after weapon burial) and October 2015 (also after weapon burial). Measurements in June and August 2012 were done using a coarse grid of 0.5 m by 1 m while that in October 2015 was done using a fine grid of 0.25 m by 0.10 m. A different coordinate system was used for measurements in 2015 compared to that in 2012. A schematic presentation for both coordinate systems is presented in Fig. 4.
Data source location	Institution: University of Toronto City/Town/Region: Toronto, Ontario Country: Canada Latitude and longitude (and GPS coordinates, if possible) for collected samples/data: Data was collected in Peel County, Canada, on private property approximately 25 km NW of Pearson International Airport (YYZ); please contact the corresponding author if you need exact location.
Data accessibility	Repository name: Mendeley Data Data identification number: DOI: 10.17632/wpcm7ffyjn.2 Direct URL to data: https://data.mendeley.com/datasets/wpcm7ffyjn/2 Instructions for accessing these data: n/a
Related research article	E. A. Deng, K. O. Doro, C.-G. Bank, Suitability of magnetometry to detect clandestine buried firearms from a controlled field site and numerical modeling, Forensic Sci. Int., 314, 2020, 110396. https://doi.org/10.1016/j.forsciint.2020.110396

Value of the Data

• As a first published magnetic gradiometer data collected above firearms buried to 1.8 m depth, this data could be used for assessing the use of magnetic gradiometry for locating buried firearms and similar objects in the shallow subsurface.
• The data can be of benefit to geophysicists, forensic scientists, law enforcement experts and other researchers looking to apply geophysical methods involving total and gradient magnetic fields for locating objects in the earth's shallow subsurface.
• The data may be useful as comparator for other forensic field sites, and to develop new analysis or automated algorithms for weapons searches.
• The data can also be helpful for law enforcement agents to learn about the magnetic method in a forensic context.
• The forward modeling MATLAB code may form the basis for an advanced algorithm (e.g. to automatically detect weapons) using magnetometer data.

## 1 Data Description

These datasets and MATLAB numerical codes support the published research article entitled “Suitability of magnetometry to detect clandestine buried firearms from a controlled field site and numerical modelling” by Deng et al [1]. The presented data, which are also publicly available on Mendeley data repository (see details and link in the specification table above), include:

(1) Raw data files in a folder named “data”. The “data” folder contains 3 magnetic raw data files (subfolders) acquired on June 8, 2012, August 23, 2012 and October 23, 2015. The magnetic raw data are saved as subfolders “20120608”, “20120823”, and “20151023” using the acquisition year, month and day respectively. Each subfolder contains the gradiometer data (with files ending *.g.txt) and base station magnetic data (with file ending *stn.txt or *.b.txt).

The gradiometer data files “01opp2.g.txt”, “08iu2.g.txt” and “01.cc1.g.txt” for the three different campaigns in June 2012, August 2012 and October 2015 contains the X (m) and Y (m) positions, total magnetic Field (nT) and the magnetic gradient (nT/m) in columns 1, 2, 3 and 4 respectively.

**Fig. 1:**
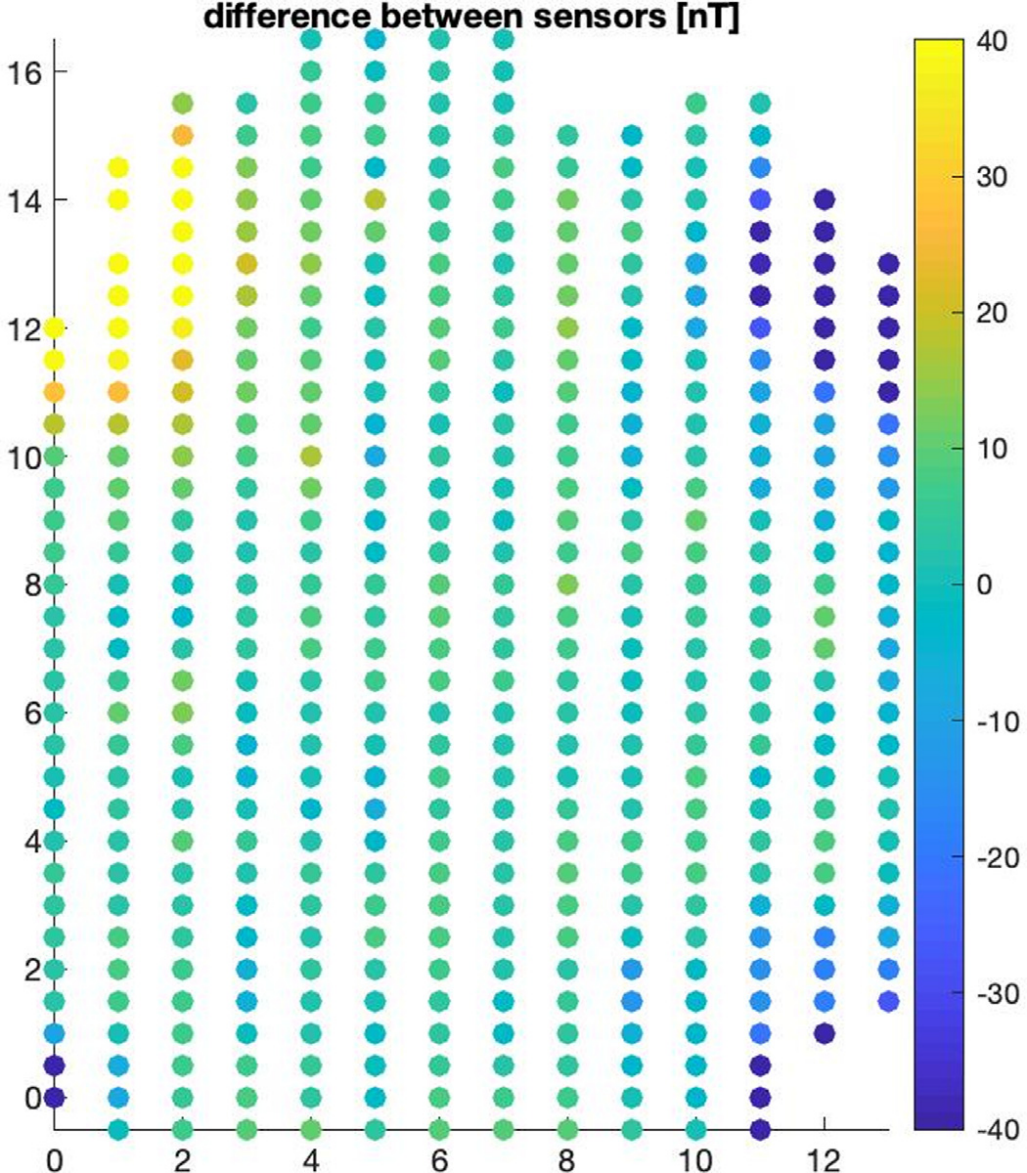
Gradiometer data collected prior to burial of weapons. Data measurement on June 8, 2012 using a 0.5 m by 1 m coarse grid. The vertical and horizontal axis represents the X and Y coordinates of the acquisition grid.

**Fig. 2:**
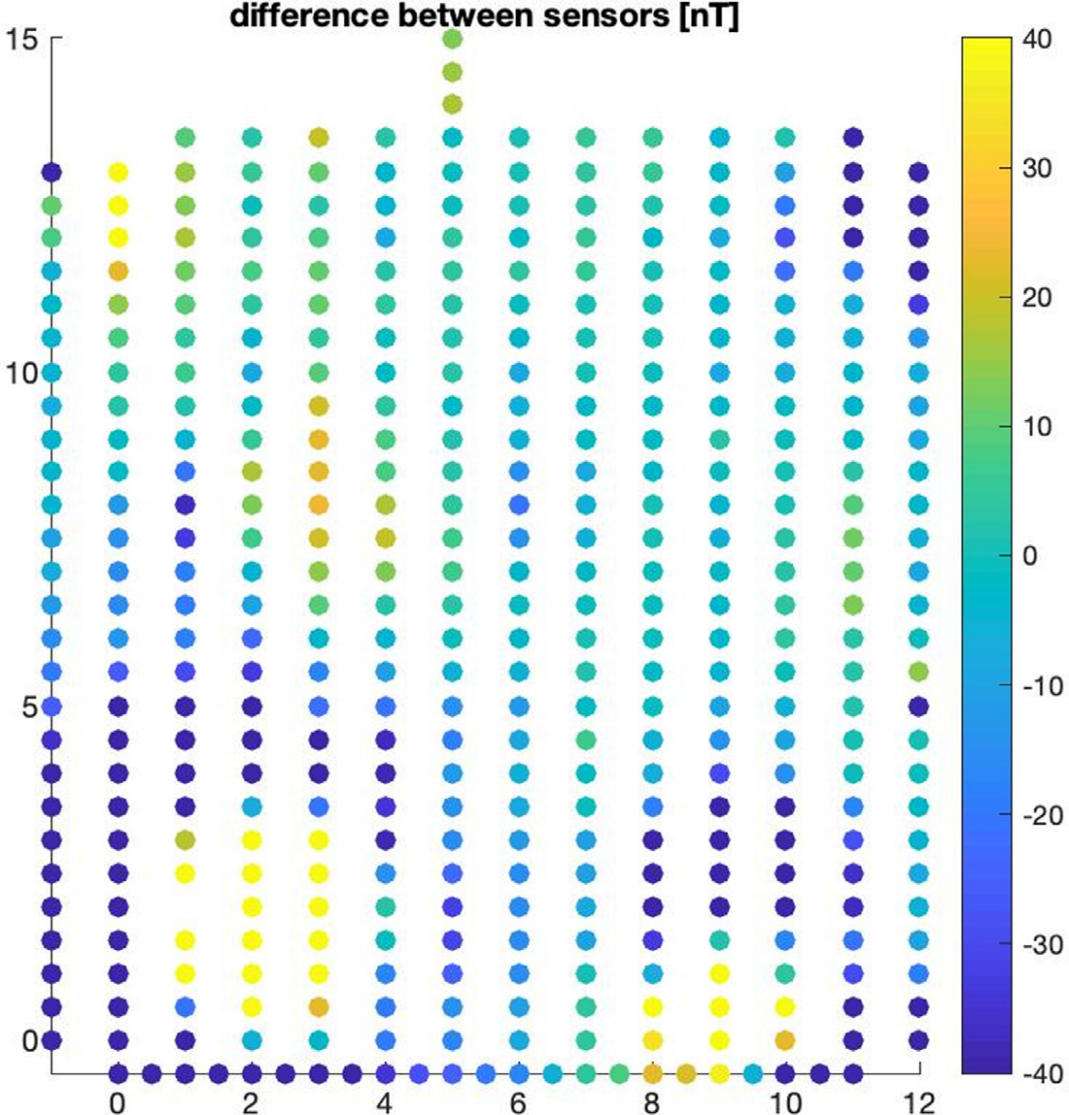
Gradiometer data collected after burial of weapons. Data measurement on August 23, 2012 using a 0.5 m by 1 m coarse grid. The vertical and horizontal axis represents the X and Y coordinates of the acquisition grid.

**Fig. 3:**
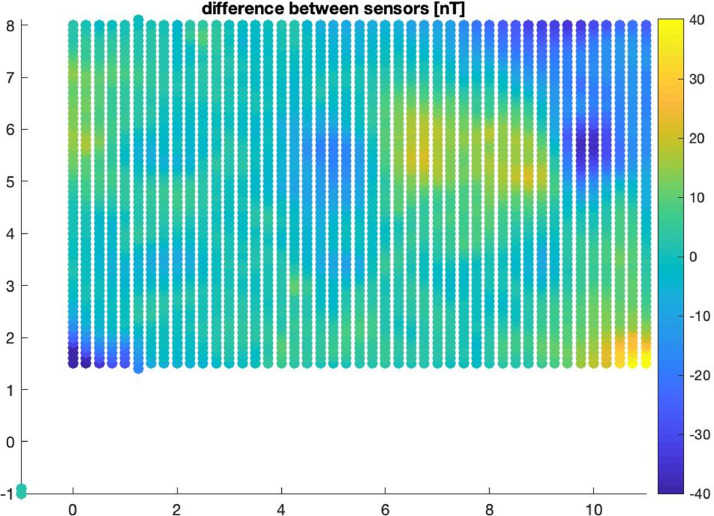
Gradiometer data collected after burial of weapons. Data measurement on October 23, 2015 using a 0.25 m by 0.10 m fine grid. The vertical and horizontal axis represents the X and Y coordinates of the acquisition grid.

The gradiometer data acquired during the 3 different measurement campaigns are also plotted in Fig. 1, Fig. 2, Fig. 3 (June 2012–Fig. 1, August 2101–Fig. 2 and October 2015–Fig. 3) with each dot mark representing a measurement point. The data folder also contains a read me file named “README_data.txt” with details on the acquired datasets.

(2) MATLAB scripts in a folder named “Matlab codes”. The MATLAB scripts are used for forward simulation of total magnetic fields and magnetic gradients response from buried firearms. The folder contains 4 scripts and 3 subfolders with their description given below.

*MATLAB scripts in parent folder “Matlab codes”*

(i) viewmagdata.m: this displays all field data.
(ii) mag_fielddata.m: this displays the data collected in October 2015 on fine grid, switches the coordinates from (0,0) at the Eastern corner to one that is more aligned to the North and extracts data profiles across the buried handguns and rifles.
(iii) modelmagmap.m: this calculates the synthetic magnetic anomalies for the 6 buried weapons.
(iv) mag_compare.m: this compares the synthetic to field data, plot maps of the data, model, and their difference, plots profiles of data and model over handguns and rifles calculates the root mean square (RMS) error.

*MATLAB scripts in subfolder “modelling” in the parent folder “Matlab codes”.*

The subfolder “modeling" provides scripts to model the buried firearms using our extended long dipole approach [1] with equations presented by Seleznyova et al. [2]. The scripts include:

(i) longdipole.m: this converts B-field into cartesian coordinates
(ii) mag_Wtotal.m: calculates a map of the total magnetic field for a dipole, calls longdipole.m, does not consider background field, and it is useful if you plan to overlay several anomalies
(iii) dipolemap.m: calculates the gradient anomaly (accounting for background field), shows gradient results also calls longdipole.m

*MATLAB scripts in subfolder “matlab_scripts” in the parent folder “Matlab codes”.*

(i) G856_read2.m: to read raw magnetic base-station data for Geometrics equipment.
(ii) GEM_read_b.m: to read raw magnetic base-station data for Gem Systems equipment.
(iii) GEM_read_g.m: to read raw magnetic rover data for Gem Systems equipment.
(iv) extract_profile.m: allows user to select a profile from a gridded data set.
(v) newcolours.m: creates colour schemes (e.g., red-white-blue).
(vi) rotatemap.m: rotates a gridded map (e.g., from given direction to UTM North).

## 2 Experimental Design, Materials and Methods

**Fig. 4:**
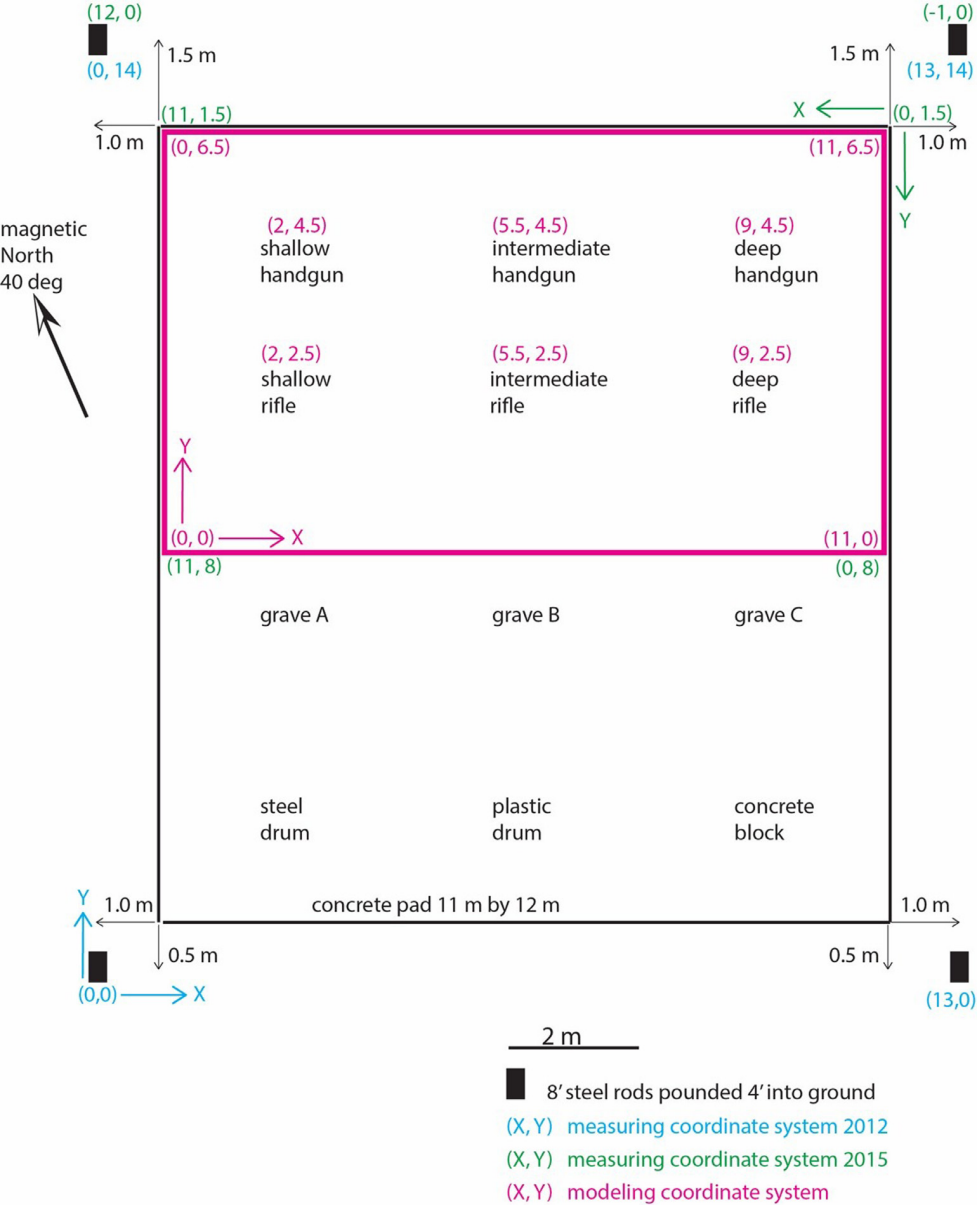
Schematic representation of the experimental domain with the highlighted section representing the area covered by a concrete slab.

The presented dataset and numerical codes are based on both field and numerical experimental design for assessing the use of gradient magnetometers for detecting buried firearms. The field test site located approximately 25 km Northwest of Pearson International Airport in Ontario, Canada is designed to advance forensic investigation of buried objects using multiple methods including geophysics [5]. The test site (Fig. 4) was prepared by burying several objects including steel and plastic drums, concrete blocks, decommissioned handguns and rifles, simulating typical objects of interest in forensic searches. The dataset presented here however, focuses on the buried handguns and rifles at varying depths of 0.6 m, 1.2 m and 1.8 m with the different burial depths used to assess the depth limitation of the presented method. A concrete slab with a 11 m x 12 m dimension was placed over the buried firearms at the site mimicking potential burial at crime sites. The six burials are separated by 2.0 m to 3.5 m (Fig. 4) to minimize interference of their magnetic signals. All firearms were placed horizontally and oriented into the same Northwest-Southeast direction.

Magnetic gradiometer data was collected prior to and after burial of the weapons. Post burial data were collected over the concrete pad placed over the weapons after burial. Magnetic measurements were done using a GEM-Systems GSM-19GW Overhauser gradiometer which measures both the total magnetic field and the vertical gradient with a nominal sensitivity of 0.0015 nT. The gradiometer consists of two magnetic sensors separated vertically by a distance of 0.55 m with the lower and upper sensors placed at elevations of 0.25 m and 0.80 m above ground level. The concrete pad covers 12 burials as shown in Fig. 4. Data collected in June and August 2012 were done using a coarse grid of 0.5 m by 1 m while that in October 2015 was done using a fine grid of 0.25 m by 0.10 m. This data-in-brief contains all the raw data and MATLAB scripts to recreate analysis of the data including figures shown in [1]. During each of the measurements with the magnetic gradiometer, a base station was also established ∼ 30 m Southeast of the measurement grid to monitor ambient changes in the magnetic field. Magnetic measurements at the base station was done using a Geometrics G-856AX portable proton magnetometer.

The numerical experimental set up involved first formulating a conceptual framework for the likely magnetic anomaly that would result from the buried weapon at the shallow subsurface (in this case, 0.6 m, 1.2 m and 1.8 m respectively). We assumed a long dipole model to represent the resultant anomaly [1]. The second step was to extend long dipole equation earlier presented by Seleznyova et al. [2], [3], [4] to represent our formulated long dipole anomaly solution applying simple victor algebra and vector superposition [1]. Finally, we coded the equations in MATLAB allowing us run a forward simulation for estimating parameters describing the anomaly including its length and magnetization, as well as the location of its center in the subsurface and the azimuth (angle to North) and plunge (angle from the horizontal).

## 3 Ethics Statement

The authors have adhered to Ethics in research and publication guidelines.

## Declaration of Competing Interest

The authors declare that they have no known competing financial interests or personal relationships which have, or could be perceived to have, influenced the work reported in this article.

## Appendix Supplementary materials

Image, application 1

## References

[1] E. A. Deng, K. O. Doro, C.-G. Bank, Suitability of magnetometry to detect clandestine buried firearms from a controlled field site and numerical modeling, Forensic Sci. Int., 314, 2020, 110396 10.1016/j.forsciint.2020.110396 32663720

[2] Seleznyova K., Strugatsky M., Kliava J. (2016). Modelling the magnetic dipole. Eur. J. Phys..

[3] Seleznyova K., Strugatsky M., Kliava J. (2016). Erratum: Modelling the magnetic dipole (2016Eur. J. Phys.37 025203). Eur. J. Phys..

[4] Seleznyova K., Strugatsky M., Kliava J. (2016). Reply to comment on ‘Modelling the magnetic dipole’. Eur. J. Phys..

[5] Pringle J.K., Ruffell A., Jervis J.R., Donnelly L., McKinley J., Hansen J., Morgan R., Pirrie D., Harrison M. (2012). The use of geoscience methods for terrestrial forensic searches. Earth-Sci. Rev..

