# Supplementary material for: Gradient magnetometer dataset and MATLAB numerical code for simulating buried firearms at a controlled field site
Source: Data Brief. 2020 Jul 21;31:106050. doi: 10.1016/j.dib.2020.106050 (PMC7393520; doi:10.1016/j.dib.2020.106050)
Supplement: Supplementary file 1 [file mmc1.zip › Notes.pdf]

Notes on magnetic data and modeling

Kennedy O. Doro and Carl-Georg Bank, 06 June 2020

1. DATA

in "data" directory, see `README_data.txt` file
`viewmagdata.m` displays field data (basemag and rover; June 2012, Aug 2012, Oct 2015)
manuscript uses 2015 dataset

detailed site setup:

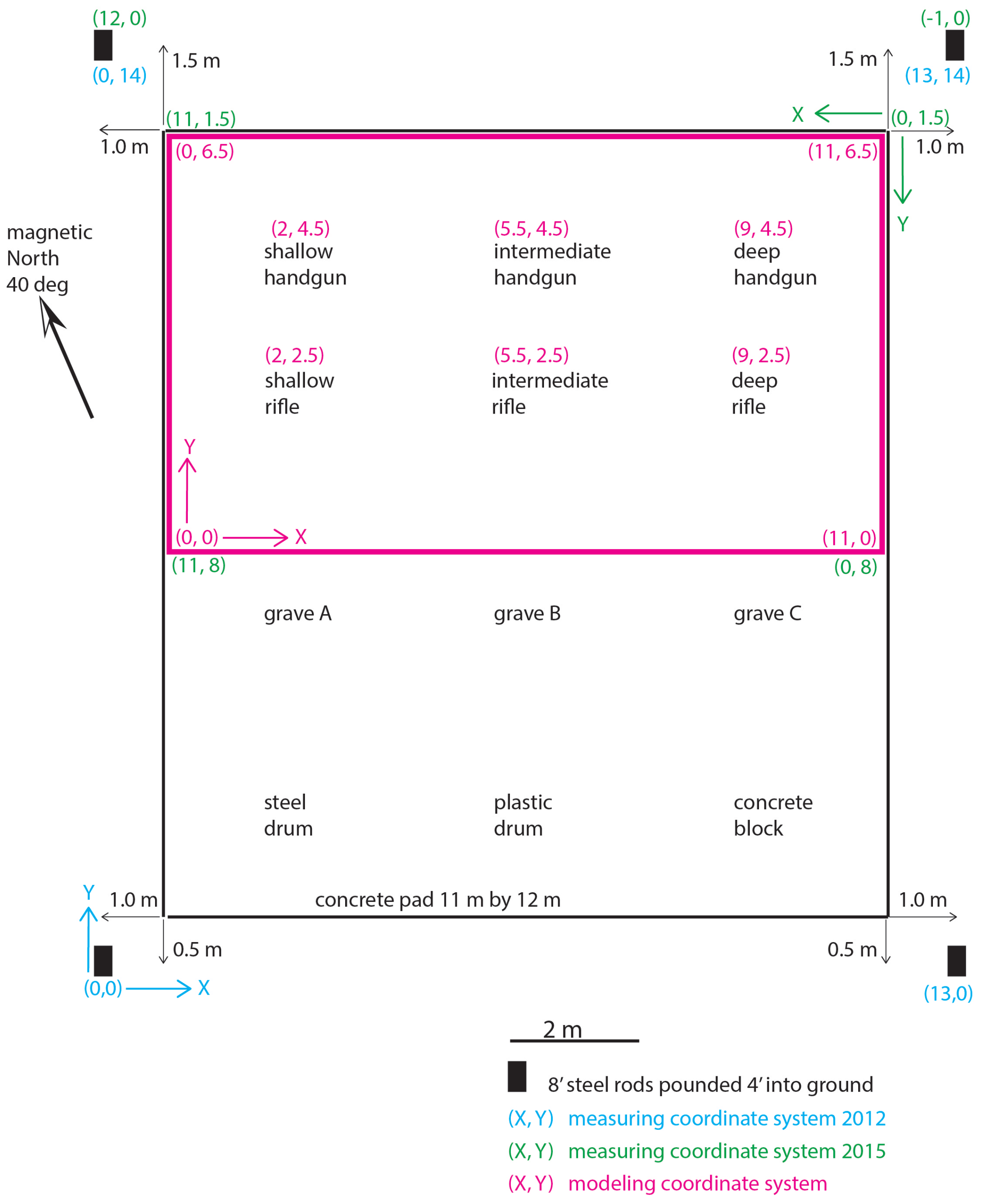

2. MODELING AND DATA ANALYSIS

to recreate data and models in manuscript run

a. `magfielddata.m`

- views data collected in Oct 2015
- switches coordinates from (0,0) at E corner to one that is more aligned to N
- extracts data profiles across guns and rifles

b. `modelmagmap.m`

- calculates synthetic anomaly for the 6 weapons (map and profiles)

c. `mag_compare.m` (will use mat files created by scripts in a and b)

- to compare synthetic and field data
- maps data, model, difference
- plots profiles of data and model over handguns and rifles
- calculates rms fit

directory "mat_files" contains our output from magfielddata.m and modelmagmap.m so one can run mag_compare.m

directory "modeling"

`longdipole.m`

- converts B-field into cartesian coordinates using formula by Seleznyova et al., 2016

`mag_Wtotal.m`

- calculates map of total field for long dipole
- calls longdipole, does NOT consider background field
- useful if you plan to overlay several anomalies

`dipolemap.m`

- calculates gradient anomaly for that dipole (with background field),
- shows gradient results on a map
- also calls longdipole

directory "matlab_scripts"

`G*_read*.m`	read data files saved from Geometrics G856 and GEM magnetometers
`extract_profile.m`	allows user to take profile from a gridded data set
`newcolours.m`	to create colour schemes, ex: divergent red-blue, sequential light to dark blue, categorial
`rotatemap.m`	rotates a gridded map
