# Supplementary figures and images for: Gradient magnetometer dataset and MATLAB numerical code for simulating buried firearms at a controlled field site
Source: Data Brief. 2020 Jul 21;31:106050. doi: 10.1016/j.dib.2020.106050 (PMC7393520; doi:10.1016/j.dib.2020.106050)

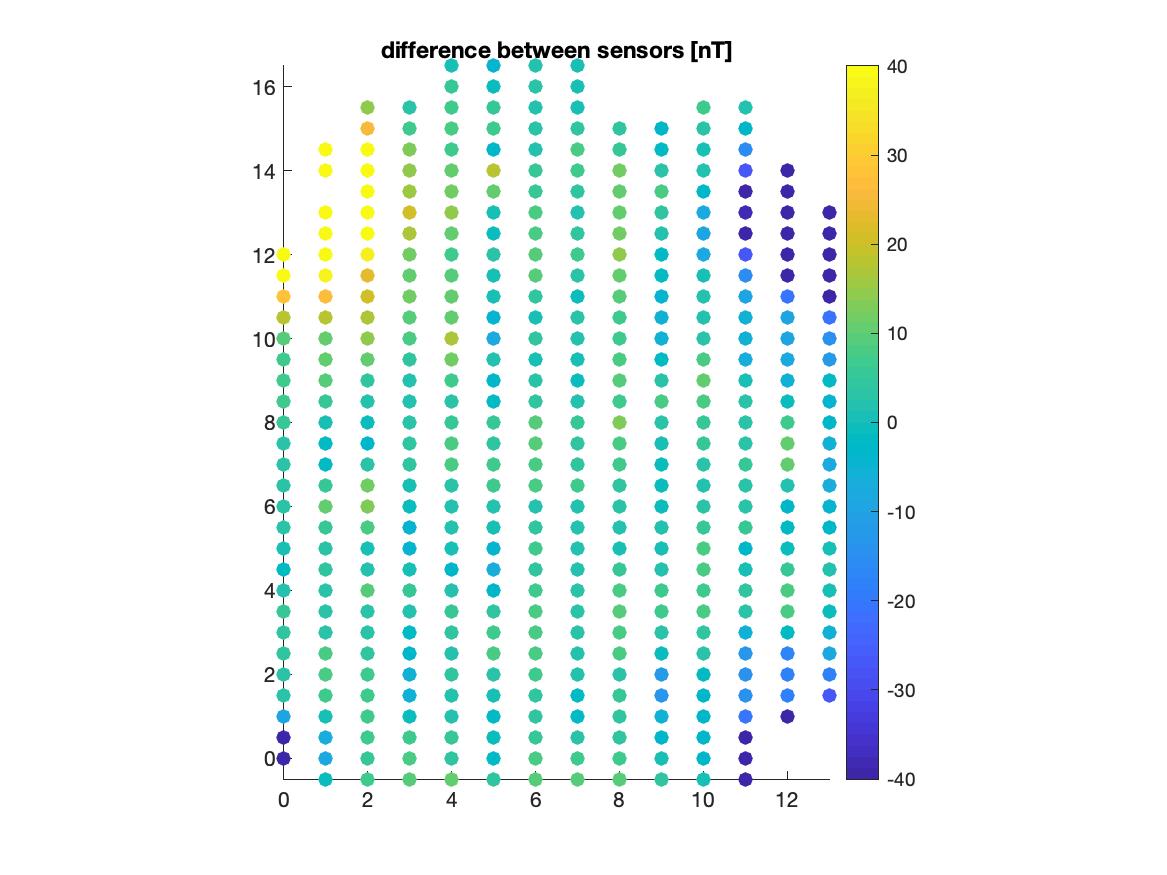

Supplement: Supplementary file 1 [file mmc1.zip › data/2012-06-difference.jpg]

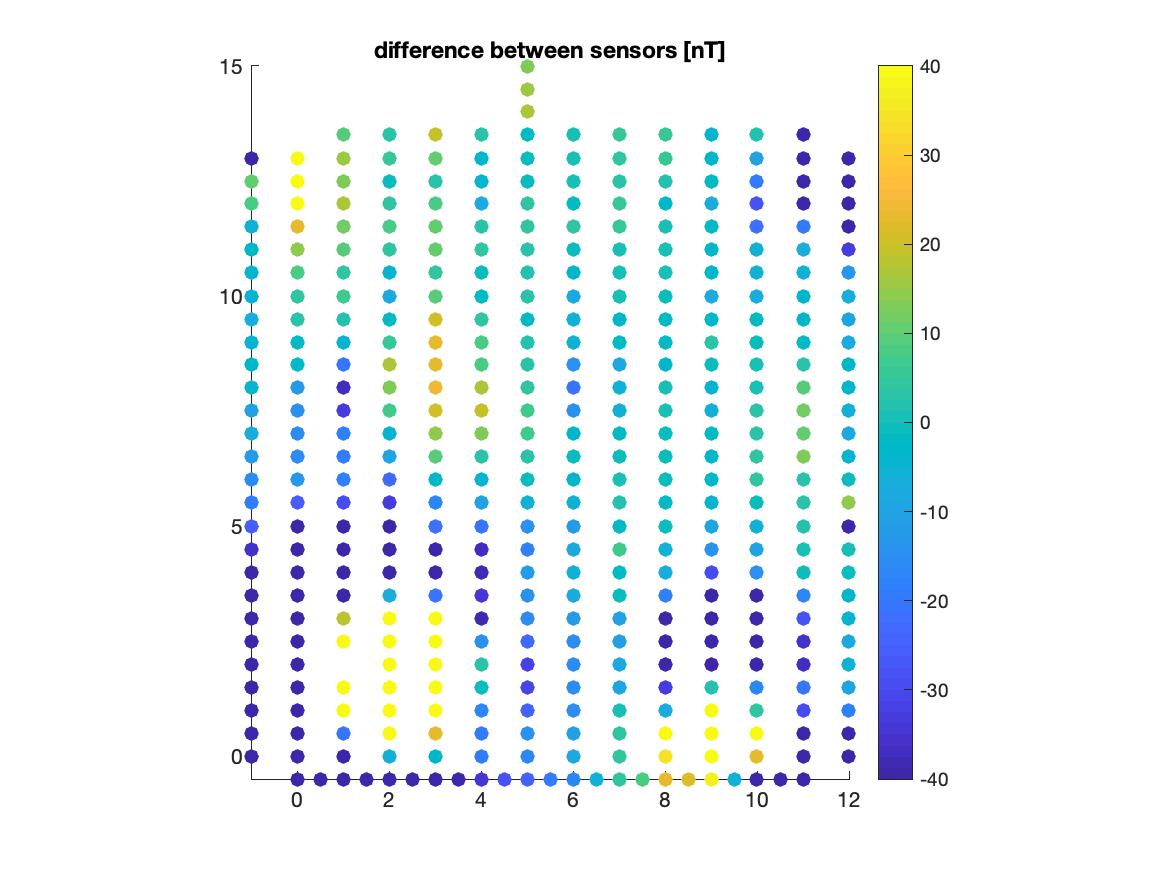

Supplement: Supplementary file 1 [file mmc1.zip › data/2012-08-difference.jpg]

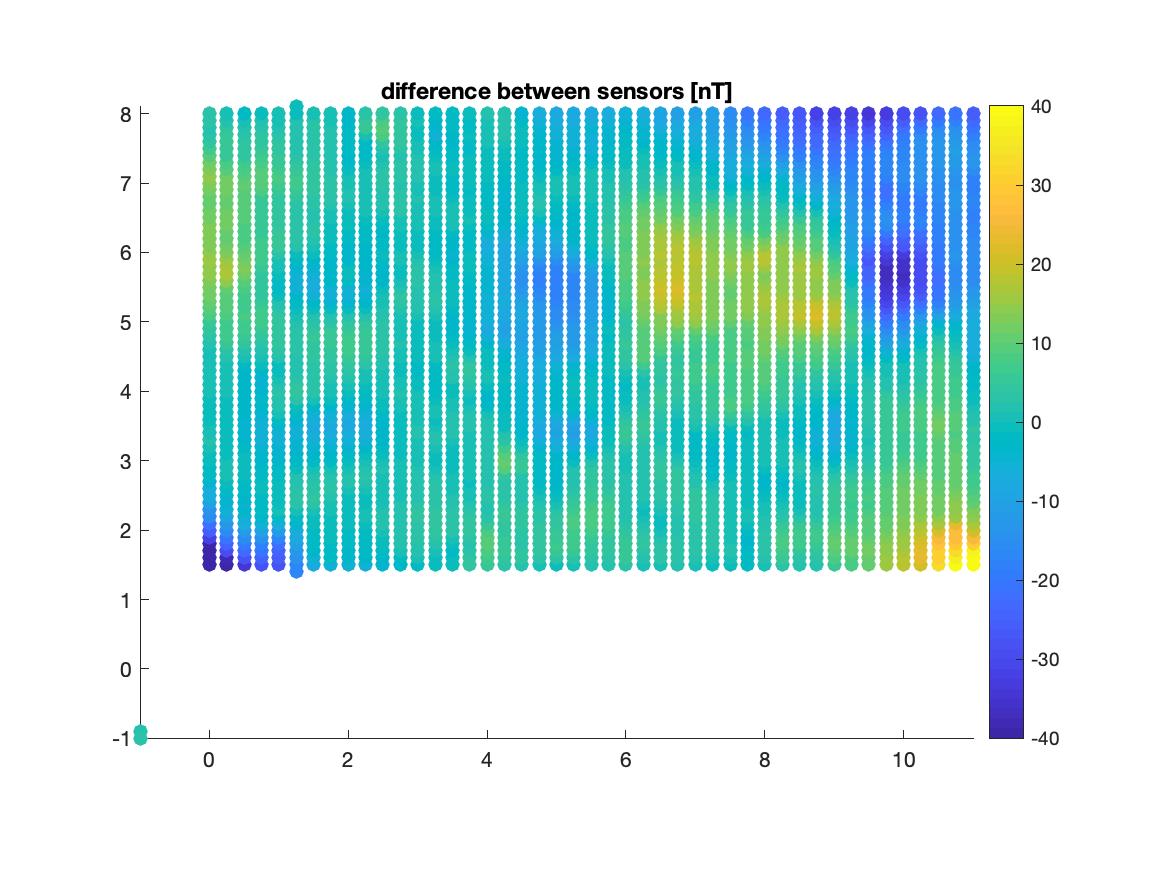

Supplement: Supplementary file 1 [file mmc1.zip › data/2015-10-difference.jpg]
